# Is There an Association Between Cesarean Section Delivery with Specific Learning Disabilities (SLD) or/and Attention-Deficit/Hyperactivity Disorder (ADHD)? A Cross-Sectional Study in Greek Population

**DOI:** 10.3390/children11111386

**Published:** 2024-11-14

**Authors:** Maria A. Makri, Dimitrios Chaniotis, Victoria G. Vivilaki, Effie G. Papageorgiou

**Affiliations:** 1 Department of Biomedical Sciences, University of West Attica, Agiou Spiridonos 28, Egaleo, 12243 Athens, Greece; dchaniotis@uniwa.gr (D.C.); efipapag@uniwa.gr (E.G.P.); 2 Midwifery Department, University of West Attica, Agiou Spiridonos 28, Egaleo, 12243 Athens, Greece; vvivilaki@uniwa.gr

**Keywords:** specific learning disabilities, attention-deficit/hyperactivity disorder, mode of delivery, comorbidity, cesarean section, vaginal birth, birthweight, gestational age

## Abstract

Background/Objective: Learning difficulties (LDs) are lifelong neurodevelopmental disorders with multifactorial causes, including perinatal factors like mode of delivery. This study aims to explore whether cesarean section (CS) delivery is linked to the occurrence of specific learning disabilities (SLDs), attention-deficit/hyperactivity disorder (ADHD), or their comorbidity. Methods: An online questionnaire was distributed via Google Forms to Greek mothers and parents of children with and without diagnoses, shared through school-related groups and various Greek pages focused on child development, special education, and learning difficulties. Conducted over eight months (October 2023–May 2024), this cross-sectional study involved 256 children, 137 with LDs diagnoses, and 119 controls. Results: In total, 59.9% of CS-born children had a diagnosis, compared to 40.1% of those born vaginally (X²(1) = 4.19, *p* = 0.045). CS delivery was associated with a 68% increased likelihood of LDs (OR = 1.68, 95% CI [1.02, 2.76]), with higher risks for ADHD (OR = 2.25, 95% CI [1.06, 4.79]) and comorbid SLD/ADHD diagnoses (OR = 2.75, 95% CI [1.17, 6.46]). Stratified analyses showed birthweight and gestational age as effect modifiers rather than confounders. Key postnatal risk factors identified were family history (OR = 4.65, 95% CI [2.41, 8.94]) and language acquisition difficulties (OR = 5.28, 95% CI [1.36, 20.47]). Conclusions: The findings suggest a possible association between CS and LDs, along with a novel link between CS and increased comorbidities. These results underscore the need for further research and provide valuable insights into how CS delivery may influence the risk of LDs, depending on the type of diagnosis.

## 1. Introduction

Learning difficulties (LDs) have a neurological basis and significantly impede the learning and processing of information. They are classified as neurodevelopmental disorders that lead to deficits, significantly interfering with academic achievement, occupational performance, and activities of daily life [1]. Dyslexia, dyspraxia, dyscalculia, etc. are specific learning disabilities (SLDs) that usually coexist with ADHD. According to the fifth edition of the Diagnostic and Statistical Manual of Mental Disorders (DSM-V), ADHD is also a neurodevelopmental disorder characterized by significant levels of inattention, disorganization, and/or hyperactivity–impulsivity. Inattention and disorganization manifest as difficulty staying focused on tasks, appearing inattentive, and frequently losing materials, all of which surpass expected levels for one’s age or developmental stage. Hyperactivity–impulsivity is marked by excessive movement, restlessness, difficulty remaining seated, interrupting others, and impatience—symptoms that exceed typical behaviors for an individual’s age or developmental level [2].

SLDs affects and more specifically, decrease school and academic performance, because they cause difficulties in one or more of the following sections: in the learning or comprehending process, in oral communication, in writing or written expression, in reading skills, in the perception or calculation of the numbers, and in mathematic problem solving [2]. These difficulties cannot be attributed to language deficiency, mental retardation, sensory loss, and other psychiatric, neurological, or psychosocial conditions. They also cannot be explained by low intelligence functioning as students with SLD have at least a normal level of intelligence quotient [3]. As a result, SLD is described as the inability to achieve grade-level expectations in one or more of the aforementioned areas, despite the availability and the provision of learning opportunities according to each child’s state and age [4].

The prevalence of SLDs in reading, writing, and mathematics ranges from 5% to 15% among school-age children, but rates can vary due to sample size, inclusion criteria, and definitions [2,5,6]. Diagnoses are more common in boys than in girls, with ratios of 2:1 to 3:1, though some studies report different gender differences based on specific deficits [2,5]. Notably, ADHD is the most common comorbidity among children with SLD [7,8]. However, the broadening of ADHD diagnostic criteria in DSM-5 raises concerns about overdiagnosis, as typical childhood behaviors may be pathologized. This highlights the need for caution in the diagnostic process, as the subjective nature of ADHD symptoms and their overlap with other conditions can lead to misdiagnosis, complicating the evaluation of children with other difficulties [9,10].

Although the exact causes of SLD and ADHD are not yet fully understood, numerous risk factors have been identified. Firstly, both SLD and ADHD are familial and heritable [11]. Specific learning disabilities tend to run in families [12,13]. The risk of these disorders is significantly higher in first-degree relatives of affected individuals, with a 4–8 times greater risk for reading disorders and a 5–10 times greater risk for mathematics disorders compared to those without such difficulties [2]. Accordingly, ADHD is more prevalent among biological relatives of individuals with ADHD, indicating a substantial heritability based on genetics [14,15]. Other etiological factors are associated with the function of the brain and the neural structure [16,17,18].

Environmental factors, alongside heritability and genetics, significantly influence individual and brain development. Specifically, ADHD is also influenced by several key factors affecting social interactions and development. Individuals with ADHD often face difficulties in social cognition, which hampers their ability to recognize emotions and interpret social cues, complicating their relationship-building efforts [19]. A poor linguistic family environment can exacerbate these issues, as children may experience delays in language development, impacting their processing and organizational skills [20,21]. Additionally, increased use of electronic devices contributes to attention problems and reduces meaningful face-to-face interactions [22]. Lack of play opportunities further limits the development of critical social skills like impulse control and emotional regulation [23]. Overall, promoting a rich linguistic and interactive environment is crucial for reducing ADHD-related risks, particularly for children in under-stimulating contexts. This highlights the need for comprehensive approaches to understanding and managing ADHD, addressing both social and developmental aspects.

Other environmental factors are related to many prenatal, perinatal, and postnatal characteristics and may contribute to SLD or ADHD. According to recent research, several strong causative factors associated with the above disorders include tobacco smoke [24,25], toxic agents, environmental contaminants and maternal infections [26], maternal [27] and parental age [28], prematurity [29,30,31,32] and low birthweight [33,34,35], lack of breastfeeding [36,37], head injuries, and complications during pregnancy or childbirth [26].

Additionally, CS has been identified as an environmental perinatal risk factor for neurodevelopmental disorders like ADHD [38,39,40], as well as for some difficulties in reading accuracy and phonetic and visual orthography [41]. There is also evidence suggesting an association between CS and cognitive performance [42,43,44]. However, according to Blake et al.’s systematic review, the correlation between CS and cognitive functioning in offspring remains inconsistent [45]. Furthermore, infants delivered by CS have an increased risk of various health problems [40,46,47,48,49,50]. Considering all these associations and connections, concerns about the rising global rates of CS are justifiably heightened. 

In conclusion, as mentioned, CS has been associated with various problems, disorders, and difficulties. However, some of these associations remain controversial, since different scientific studies around the world have reached varying conclusions. Notably, in Greece, where the rate of cesarean births is particularly high [51], the potential effects of CS on children’s brain, cognitive, and learning development have not been investigated. As a result, it remains unclear whether CS delivery increases the likelihood of SLD, ADHD, or comorbidity diagnosis involving both SLD and ADHD. This study’s primary contribution is its exploration of the association between CS delivery and various diagnoses, including neurodevelopmental disorders such as SLD and ADHD, and their comorbidity in the Greek population. 

## 2. Materials and Methods

### 2.1. Study Participants 

The Ethics Committee of the University of West Attica approved our study protocol (Reference Number: 51,324, dated 26 May 2023). The study was conducted in accordance with the ethical standards of the Helsinki Declaration.

An online questionnaire was created using Google Forms to survey Greek mothers or parents of children, both with and without a diagnosis. The survey was shared within Greek school-related groups. Additionally, it was posted on various Greek group pages that focus on child development, special education, and learning difficulties (LDs), attracting members from all regions of Greece. These parent groups were reputable and officially recognized, particularly sensitized to issues related to child development and education. The questionnaire remained accessible for an eight-month period, from 1 October 2023 to 1 May 2024.

Children with autism spectrum disorder (ASD) or other disabilities, except for SLD and ADHD, were excluded, along with those conceived via in vitro fertilization or other assisted reproductive technologies. In the control group without a diagnosis, participants with low school performance were also excluded to mitigate the inclusion of children potentially experiencing undiagnosed difficulties. Participation was anonymous and voluntary. The survey consisted of 48 questions, which included both fill-in-the-blank and multiple-choice formats.

### 2.2. Questionnaire for Parents

Initially, sociodemographic characteristics (age, gender, educational level, family status and income, area of residence, number of children, etc.) were collected. Then, some questions were addressed to gather information about the medical diagnoses and expert evaluations concerning the child’s difficulties. In the study group of children facing difficulties, the parents’ feedback was based on confirmed official diagnoses from recognized Greek institutions, including KEDASY (Centers for Educational and Counseling Support), private developmental specialists, and public hospital doctors, all of which are documented in the children’s medical records, ensuring the validity and undisputed accuracy of these diagnoses. Regarding students without a diagnosis (control group), some information was requested about their grades and school performance.

Furthermore, a set of questions was designed to accumulate comprehensive information about prenatal, perinatal, and postnatal events. These questions focused on collecting data about the mother’s pregnancy period (mode of conception, age of parents at conception, problems during pregnancy, illnesses, medication, smoking, preeclampsia, etc.), characteristics of childbirth (mode of delivery, rupture of membranes, mode of anesthesia, complications during childbirth, etc.) and the newborn (gestational age, weight of birth, etc.), and postnatal events (postpartum care, breastfeeding, head injuries, anesthesia for surgery, serious illnesses, etc.) that may influence children’s development. Finally, the last questions aimed to gather information on the child’s language development (language delay, difficulties in language acquisition, speech therapy) and identify whether there is a family history of a similar diagnosis.

### 2.3. Statistical Analysis

Statistical analysis was conducted using IBM SPSS 29 statistical software (SPSS Inc., Chicago, IL, USA). The continuous variables were expressed using mean ± standard deviation (mean ± SD), and the categorical variables were expressed using frequencies (N) and percentages (%). Chi-square (*X²*) tests were employed to identify differences and reveal potential associations between categorical characteristics and diagnosed or non-diagnosed cases. 

The Kolmogorov–Smirnov’s normality test was used for assessing the normality of the distribution of continuous variables. In cases of non-normally distributed variables, nonparametric Mann–Whitney *U* tests or Kruskal–Wallis tests were used instead. In the normally distributed cases, *t*-test for independent samples was used to determine any differences.

To assess the association between CS and LDs, the unadjusted odds ratios were estimated, along with 95% confidence intervals (95% CI). Further analysis was conducted using stratification. Stratification was employed to control for confounding during data analysis. This technique serves as a preliminary step for applying methods such as the Mantel–Haenszel formula and standardization. In this investigation, stratification was used to assess whether CS is causally associated with the increased frequency of LDs, independent of the confounding effects of birthweight and gestational age. 

During stratification, three subgroups were created and defined by diagnosis (SLD, ADHD, and comorbidity). Two stratified analyses were conducted based on the variable being assessed in each case. When stratified by birthweight, one group consisted of children with low birthweight (<2500 g), while the other group consisted of children with normal birthweight (>2500 g). Similarly, when stratified by gestational age, one group included preterm infants born before 36 weeks of gestation, and the other group consisted of full-term infants born after 36 weeks. For the purposes of the stratified analysis, the quantitative data on birthweight and gestational age from the survey were converted into categorical variables.

Finally, binary logistic regression was conducted to evaluate the impact of postnatal and hereditary factors on the occurrence of LDs. The model included variables such as breastfeeding, language delay, difficulties in language acquisition, and family history of LDs. These factors were selected for the regression analysis based on their significant associations with LDs in the chi-square tests.

All hypothesis tests were conducted assuming a 0.05 significance level and a two-sided alternative hypothesis.

## 3. Results

### 3.1. Sample Demographic Characteristics

In total, data of two-hundred fifty-six (256) children were successfully collected: one hundred thirty-seven (137) diagnosed cases and one hundred nineteen (119) cases without diagnosis. One hundred thirty-seven (137) students were males (53.5%) and one hundred nineteen (119) females (46.5%). Eighty-eight (88) children attend primary grades A to C (34.4%), ninety-six (96) attend primary grades D to F (37.5%), and seventy-two (72) children attend secondary grades A to C (28.1). Other parental demographic characteristics are shown in Table 1. 

### 3.2. Associations of Prenatal and Perinatal Events with Diagnosis

The present survey revealed statistically significant differences between certain perinatal events and diagnosed cases. Specifically, the prevalence of a diagnosis in children differed significantly between those born by CS (59.9%) and those born by vaginal delivery (40.1%) *X*^2^ (1) = 4.19, *p* = 0.045. Children born by CS were more likely than children born by vaginal birth to have a diagnosis of SLD, ADHD, or comorbidity.

A statistically significant correlation was also found between diagnoses and gestational weeks. The diagnosed cases (Mdn = 38.20, IQR = 2.3) present a statistically significant difference from the cases of children without a diagnosis (Mdn = 38.50, IQR = 2.0) in terms of gestational weeks (U = 6592, z = −2.65, *p* = 0.008, r = −0.17). Alternatively, as gestational age decreases, the likelihood of receiving a diagnosis increases. Furthermore, a statistically significant association was found regarding birthweight (*p* = 0.043). This indicates that children with diagnosis have statistically significant lower birthweight (M = 3068, SD = 575) in comparison with children without a diagnosis (M = 3206, SD = 499).

Finally, an association was found regarding the mode of anesthesia *X*^2^ (2) = 6.22, *p* = 0.043. Specifically, a statistically significant relationship was observed in participants where no anesthesia was used, indicating that children born to mothers who did not use anesthesia during labor are less likely to be diagnosed with difficulty.

There were no statistically significant differences in maternal or paternal ages in our sample. Additionally, no associations were found with maternal characteristics such as smoking status, pregnancy complications, and preeclampsia, or with other perinatal characteristics, including the type of CS and the mode of induction of labor (*p* > 0.05). More information about prenatal and perinatal characteristics is shown in Table 2. 

### 3.3. Associations of Other Characteristics with Diagnosis

A statistically significant association was found between both cases (diagnosed and non-diagnosed cases) and the family history of learning difficulties *X*^2^ (1) = 26.37, *p* < 0.001. Specifically, among children with a family history of LD, 60 (77.9%) received a similar diagnosis, compared to only 17 (22.1%) among those without a family history. This result underscores the strong relationship between family heredity and the likelihood of a learning difficulty diagnosis. 

Additionally, a statistically significant relationship was found between all cases and both difficulties *X*^2^ (1) = 23.19, *p* < 0.001, and delays *X*^2^ (1) = 22.83, *p* < 0.001 in language acquisition. Among all children with language acquisition difficulties, 34 children (89.5%) had been diagnosed with a disability. Furthermore, among all children with a language delay, 47 children (81%) had such a diagnosis. Similarly, statistically significant relationships were found between both cases and speech therapy *X*^2^ (1) = 40.63, *p* < 0.001 as well as postpartum care *X*^2^ (1) = 11.63, *p* = 0.001. 

Finally, breastfeeding was associated with lower rates of any diagnosis, *X*^2^ (1) = 5.84, *p* < 0.018, indicating that non-breastfed children are more likely to be diagnosed with SLD, ADHD, or comorbidity. Among all non-breastfed children, 35 (68.6%) had a diagnosis, compared to only 16 (31.4%) who were not diagnosed. The breastfeeding period included in our sample was defined as a minimum of one month of exclusive breastfeeding. More information about these associations is shown in Table 3.

### 3.4. Stratified Analysis

#### 3.4.1. Stratification Results According to Birthweight

According to the unadjusted results, children born via CS have 1.68 times higher odds of developing learning disabilities compared to those born vaginally, indicating a 68% increased likelihood of developing LDs for children born via CS. This odds ratio is statistically significant at the 5% level (95% CI: 1.02–2.76, *p* = 0.041), as the confidence interval does not include the null value of 1. 

In the stratified analysis by birthweight, among children with normal birthweight, those born via CS have 1.60 times the odds of developing LDs compared to those born vaginally, but this association is not statistically significant (95% CI: 0.94–2.72, *p* = 0.079). In children with low birthweight, the odds ratio is 0.95 (95% CI: 0.08–11.80, *p* = 0.968), indicating no significant association, too. The two stratum-specific odds ratios differ notably, suggesting evidence of interaction, with birthweight acting as an effect modifier rather than a confounding factor.

Moreover, the Mantel–Haenszel adjusted odds ratio is 1.57, indicating that after adjusting for birthweight, children born via CS still have 1.57 times higher odds of developing LDs compared to those born vaginally. However, this adjusted odds ratio is not statistically significant according to the Cochran–Mantel–Haenszel test (*p* = 0.088). Since the unadjusted and adjusted odds ratios differ by less than 10%, birthweight does not appear to be a confounding factor in this analysis. 

The same result is confirmed in the stratified analysis by birthweight within subgroups defined by diagnosis, as shown in Table 4. In the SLD group, where stratum 1 has an undefined odds ratio, it is unclear whether there is evidence of interaction. However, since the unadjusted and adjusted odds ratios do not differ significantly, there is no evidence of confounding in this group.

In the ADHD group, low birthweight also acts as an effect modifier because the stratum-specific odds ratios differ from one another, and the unadjusted odds ratio falls between them. Based on the adjusted results in this group, children born via CS have 2.08 times higher odds of developing ADHD, compared to those born vaginally.

In the comorbidity group, the undefined odds ratio in stratum 1 makes it unclear whether there is evidence of interaction, too. However, this is the only group where there is some evidence of confounding, as the unadjusted and adjusted odds ratios differ by more than 10%.

#### 3.4.2. Stratification Results According to Gestational Age

In the stratified analysis by gestational age, among children born at normal gestational age, those delivered via CS have 1.54 times higher odds of developing LDs compared to those born vaginally. However, this association is not statistically significant (95% CI: 0.92–2.57, *p* = 0.096). In premature children, the odds ratio is 3.67 (95% CI: 0.35–38.03, *p* = 0.261), also indicating no significant association. The notable difference between the two stratum-specific odds ratios suggests potential interaction, with gestational age acting as an effect modifier rather than a confounding factor.

Additionally, the Mantel–Haenszel adjusted odds ratio is 1.60, showing that after adjusting for gestational age, children born via CS still have 1.60 times higher odds of developing LDs compared to those born vaginally, representing a 60% increased likelihood. However, this adjusted odds ratio is not statistically significant (*p* = 0.065) according to the Cochran–Mantel–Haenszel test. As the unadjusted and adjusted odds ratios differ by less than 10%, gestational age does not appear to be a confounding factor in this analysis, suggesting that CS may be associated with an increased risk of developing LDs.

The same result is confirmed in the stratified analysis by gestational age within subgroups defined by diagnosis, as shown in Table 5. In the SLD group, the unadjusted and adjusted odds ratios do not differ significantly, indicating that gestational age is not a confounding factor. However, in this subgroup, CS does not appear to be associated with the development of SLDs (OR = 1.16, 95% CI: 0.64–2.10, *p* = 0.629).

In the ADHD group, gestational age acts as an effect modifier, as the stratum-specific odds ratios differ, and the unadjusted odds ratio lies between them. Based on the adjusted results, children born via CS have 2.37 times higher odds of developing ADHD compared to those born vaginally, representing a 137% increased likelihood of developing ADHD (95% CI: 1.09–5.18, *p* = 0.030).

In the comorbidity group, the undefined odds ratio in stratum 1 also makes it unclear whether there is evidence of interaction. As a result, gestational age again acts as an effect modifier. Notably, children born via CS have 2.33 times higher odds of developing both SLD and ADHD compared to those born vaginally, meaning that CS children have a 133% increased likelihood of dealing with comorbidity conditions (95% CI: 0.99–5.46, *p* = 0.048).

### 3.5. Associations Between Possible Influencing Postnatal and Hereditary Factors with Diagnosis 

In light of the stratified analysis results, which demonstrated an association between CS and LDs, and indicated that birthweight and gestational age serve as effect modifiers rather than confounders, we conducted a logistic regression model using the forward stepwise Wald method. The aim of our model was to explore the actual relationship between CS and LDs while accounting for the influence of a family history of LDs and other additional environmental factors such as breastfeeding, language acquisition difficulties, and language delay.

The model was statistically significant, X² (3) = 55.814, *p* < 0.001, and correctly classified 69% of the cases. In this model, the association between CS and LDs weakened. However, family history of LDs (OR = 4.662, 95% CI [2.460, 8.837]) and language acquisition difficulties during the preschool or school period (OR = 9.798, 95% CI [3.271, 29.347]) were strong predictors of the development of SLD, ADHD, or their comorbidity. Table 6 presents the unadjusted results and additional information on the regression model.

## 4. Discussion

Learning disabilities undoubtedly pose significant risks to a child’s physical and mental health. As a multifactorial disorder, it is essential to consider environmental influences alongside genetic factors. The objective of this study was to analyze the association between an environmental factor, cesarean section delivery (CS), with the diagnosis of specific learning disabilities (SLD), attention-deficit/hyperactivity disorder (ADHD), and their comorbidity evaluating the degree to which type of delivery may be related to the risk of having difficulties. 

As a result, the prevalence of these difficulties was higher in children born via CS compared to those born vaginally. In our sample, the type of delivery was related statistically to the disabilities found, constituting a risk factor. Regarding the results in the subgroups of stratified analysis, it is important to note that statistically significant relationships were found in subgroup ADHD, and in comorbidity subgroup, concerning cases where SLD coexists with ADHD. More specifically, in our study children born via CS are more likely to be diagnosed with ADHD or with comorbidity. These results align with other studies that revealed statistically significant correlations between ADHD and cesarean births [38,39,40]. The strong association observed between CS and the rate of comorbidity diagnoses is a novel finding, suggesting that different causative factors may contribute to both specific learning disabilities (SLD) and ADHD. The lack of a statistically significant relationship between cesarean births and cases involving only SLD indicates that these distinct difficulties require further investigation. Based on the above results, CS appears to be causally linked to an increased occurrence of ADHD. However, the absence of a direct association between CS and SLDs suggests that other factors are likely responsible for these disabilities. Consequently, children with SLDs may be more prone to being diagnosed with comorbidity conditions, if they were born via CS. 

Furthermore, in our sample, gestational age emerged as a risk factor for developing difficulties, in line with findings from other studies that report that preterm infants (born under 36 gestational weeks—gestational age) have an elevated risk of developmental delay [29,30,31]. In addition, according to a similar study mental development increases for each additional week of gestation [32]. Our study also reveals an association between birthweight and diagnosis rates, aligning with other studies that state that low birthweight is linked to learning problems, reduced cognitive abilities, and ADHD symptoms [48,49,50]. However, in our sample, the stratified analysis revealed that the above variables, birthweight and gestational age, do not act as confounders in the association between CS and LDs. Instead, they function as effect modifiers, altering the strength of the association across different populations. It should be also noted that, in our research, the gender of the child and the age of the mother or father at conception did not affect the average diagnosis rate, contrary to findings from other studies [2,27,28]. 

Our research also highlighted the heritability of the above difficulties, as most diagnosed individuals had a family member with a similar diagnosis. According to studies, both SLD and ADHD, have a hereditary basis [12,13,14,15,16,17]. Previous research also indicates that the likelihood of children developing a learning disability significantly increases if there is a family history of such conditions [52,53]. In particular, children with a family history of reading disabilities are four times more likely to develop a reading disability compared to their peers without such a history [52]. Moreover, having a positive family history has been demonstrated to be a predictive risk factor for estimating a child’s future risk of developing a disability [12,54,55]. Regarding ADHD, a recent meta-analysis of twin studies showed that the heritability of ADHD is estimated at 77–88% [15,56]. 

This study also showed that most cases of diagnoses encounter language problems during the preschool and school years. This result is consistent with recent research that has shown that early language abilities forecast individual variations in phonological awareness and letter recognition, which subsequently influence reading skills [57]. Studies also identified a direct impact of language on later word decoding abilities which indicates that broader oral language problems and challenges may be additional risk factors for learning disabilities like dyslexia [54,57,58]. Supporting this, children with specific language impairments are at a high risk of developing SLD [54,57,58] and ADHD [59,60].

Finally, in our sample, a minimum of one month of exclusive breastfeeding seems to have a positive effect on a child’s development since non-breastfed children are more likely to be diagnosed with SLD and/or ADHD. This result is consistent with other studies that associate breastfeeding with higher learning skills in school-aged children [37] and improved cognitive development [36]. Breastfeeding has well-documented benefits, and extensive support is crucial, particularly in cases of preterm births [61]. 

This study provides valuable insights for future research on how genetics and environmental factors, such as mode of delivery, may influence children’s learning processes. Specifically, the findings reveal that CS is associated with higher rates of LD diagnoses in the Greek population. As an exploratory analysis, our study identifies associations between variables and highlights patterns that warrant further inquiry, without establishing causal relationships. The results emphasize the necessity for a more structured, multidisciplinary research approach that incorporates appropriate measures and diagnostic assessments, along with comprehensive prenatal, perinatal, and postnatal data, to draw more definitive conclusions about potential correlations. Moreover, these tools should be applied individually, considering the significant influence of the socio-cultural context in which individuals develop [62], as cognitive development is a multifaceted phenomenon that requires investigation from various perspectives. Specifically, it is essential to gather, along with the full medical history, information about the child’s developmental environment, interpersonal relationships, linguistic family environment, play opportunities, and other relevant factors, since a stimulating, interactive, and supportive context can help reduce risks and provide valuable insights for a more accurate diagnosis [19,20,21,22,23].

## 5. Conclusions

This is the first study to focus on the Greek population, where CS rates notably exceed internationally accepted limits [51]. The findings of this study may provide valuable insights for future research on the impact of genetics and environmental factors, such as mode of delivery, on children’s future learning performance. More specifically, the present study found that the perinatal and environmental factor of CS is associated with higher rates of LD diagnoses in the Greek population. The stratified analysis showed that cesarean births were linked to higher rates of ADHD and comorbidity diagnoses, where both SLDs and ADHD were present, since birthweight and gestational age did not act as confounding factors in our sample. The association between CS and increased comorbidities is a novel finding, underscoring the need for further research, especially considering that isolated SLDs were not found to be associated with cesarean births. Consequently, children with SLDs may be more prone to being diagnosed with comorbidity conditions, if they were born via CS. This study confirmed findings from existing international literature, such as the significant influence of hereditary factors, while contributing new insights into the effects of CS depending on the type of diagnosis. In conclusion, future research should prioritize the thorough collection of medical histories and the assessment of individuals using diagnostic tools, while carefully considering the broader environmental context of the child’s development and upbringing. A reliable diagnosis should be based on a multidisciplinary approach to ensure its accuracy.

## Figures and Tables

**Table 1:** Associations between demographic characteristics and cases with or without diagnosis of LD.

Variables	Total (*n* = 256) N (%)	With Diagnosis (*n* = 137) N (%)	Control Group (*n* = 119) N (%)	*p*-Value ^1^
Gender				
Male	137 (53.5)	81 (59.1)	56 (47.1)	0.060
Female	119 (46.5)	56 (40.9)	63 (52.9)	
Class of child				
A–C primary school	88 (34.4)	40 (29.2)	48 (40.3)	
D–F primary school	96 (37.5)	45 (32.8)	51 (42.9)	0.001 *
A–C secondary school	72 (28.1) *	52 (38.0) *	20 (16.8) *	
Birth order				
1st child	161 (62.9)	87 (63.5)	74 (62.2)	
2nd child	78 (30.5)	39 (28.5)	39 (32.8)	0.297
3rd child	13 (5.1)	7 (5.1)	6 (5.0)	
twins	4 (1.6)	4 (2.9)	0 (0.0)	
Family status				
Married	233 (91.0) *	119 (86.9) *	114 (95.8) *	
Divorced	15 (5.9) *	13 (9.5) *	2 (1.7) *	
Widow–Widower	4 (1.6)	4 (2.9)	0 (0.0)	0.004 *
Single–Unmarried	3 (1.2)	1 (0.7)	2 (1.7)	
Single Parent	1 (0.4)	0 (0.0)	1 (0.8)	
Residence area				
Urban	205 (80.1)	110 (80.3)	95 (79.8)	
Semi-urban	38 (14.8)	20 (14.6)	18 (15.1)	1.000
Rural	13 (5.1)	7 (5.1)	6 (5.0)	
Mother’s educational level				
Low	3 (1.2)	3 (2.2)	0 (0.0)	
Medium	53 (20.7)	33 (24.1)	20 (16.8)	0.068
High	200 (78.1)	101 (73.7)	99 (83.2)	
Father’s educational level				
Low	25 (9.8)	17 (12.4)	8 (6.7)	
Medium	89 (34.8)	49 (35.8)	40 (33.6)	0.235
High	142 (55.5)	71 (51.8)	71 (59.7)	
Family income				
Low	18 (7.0)	12 (8.8)	6 (5.0)	
Medium	162 (63.3)	85 (62.0)	77 (64.7)	0.537
High	76 (29.7)	40 (29.2)	36 (30.3)	

^1^ *p*-values derived from Chi-square test. * Represents significant differences, *p* < 0.05.

**Table 2:** Associations with prenatal and perinatal characteristics.

Variables	With Diagnosis (*n* = 137) N (%)	Control Group (*n* = 119) N (%)	*p*-Value ^1^
Mother’s conception age (years), mean ± sd	31.6 ± 4.2	31.2 ± 4.2	0.366
Father’s conception age (years), mean ± sd	34.7 ± 5.8	34.4 ± 4.8	0.866
Problems during pregnancy			
Yes	18 (13.1)	13 (10.9)	0.702
No	119 (86.9)	106 (89.1)	
Smoking during pregnancy			
Yes	12 (8.8)	18 (15.1)	0.123
No	125 (91.2)	101 (84.9)	
Preeclampsia			
Yes	3 (2.2)	3 (2.5)	
No	132 (96.4)	113 (95.0)	0.896
Don’t remember	2 (1.5)	3 (2.5)	
Mode of delivery			
Vaginal birth	55 (40.1) *	63 (52.9) *	0.045 *
Cesarean section	82 (59.9) *	56 (47.1) *	
Type of cesarean			
Planned cesarean	54 (65.9)	38 (67.9)	0.856
Emergency cesarean	28 (34.1)	18 (32.1)	
Mode of induction of labor			
Natural	46 (33.6)	38 (31.9)	
Artificial	28 (20.4)	27 (22.7)	0.151
Both natural and artificial	8 (5.8)	16 (13.4)	
None	55 (40.1)	38 (31.9)	
Mode of anesthesia			
Epidural	109 (79.6)	86 (72.3)	
General	13 (9.5)	7 (5.9)	0.043 *
None	15 (10.9) *	26 (21.8) *	
Birthweight (grams), mean ± sd	3068 ± 575	3206 ± 499	0.043 *
Gestational age (weeks), mean ± sd	37.9 ± 1.9	38.6 ± 1.6	0.008 *

^1^ *p*-values derived from Chi-square test. * Represents significant differences, *p* < 0.05.

**Table 3:** Associations with other characteristics.

Variables	With Diagnosis (*n* = 137) N (%)	Control Group (*n* = 119) N (%)	*p*-Value ^1^
Family history of LDs (heredity)			
Yes	60 (43.8) **	17 (14.3) **	<0.001 **
No	77 (56.2) **	102 (85.7) **	
Breastfeeding			
Yes	102 (74.5)	103 (86.6)	0.018 *
No	35 (25.5) *	16 (13.4) *	
Preschool and school difficulties in language acquisition			
Yes	34 (24.8) **	4 (3.4) **	<0.001 **
No	103 (75.2) **	115 (96.6) **	
Delay in language acquisition			
Yes	47 (34.3) **	11 (9.2) **	<0.001 **
No	90 (65.7) **	108 (90.8) **	
Speech therapy			
Yes	76 (55.5) **	20 (16.8) **	<0.001 **
No	61 (44.5) **	99 (83.2) **	
Intensive care unit			
Yes	40 (29.2) *	14 (11.8) *	0.001 *
No	97 (70.8) *	105 (88.2) *	
Anesthesia for surgery			
Yes	23 (16.8)	14 (11.8)	0.288
No	114 (83.2)	105 (88.2)	

^1^ *p*-values derived from Chi-square test. * Represents significant differences, *p* < 0.05. ** Represents significant differences, *p* < 0.001.

**Table 4:** Odds ratios of LDs in children according to mode of delivery stratifying by birthweight.

	Unadjusted Results	Low Birthweight—Stratum 1 (*n* = 32)	Normal Birthweight—Stratum 2 (*n* = 224)	Mantel–Haenszel Analysis—Adjusted Results
Main group LDs (*n* = 256)				
OR (95% CI) *p*	1.68 (1.02–2.76) 0.041 *	0.95 (0.08–11.80) 0.968	1.60 (0.94–2.72) 0.079	1.57 (0.94–2.62) 0.088
SLD group (*n* = 186)				
OR (95% CI) *p*	1.16 (0.64–2.11) 0.629	Undefined	1.14 (0.61–2.15) 0.677	1.18 (0.63–2.21) 0.599
ADHD group (*n* = 158)				
OR (95% CI) *p*	2.25 (1.06–4.79) 0.042 *	0.20 (0.01–2.88) 0.220	2.70 (1.19–6.11) 0.015	2.08 (0.97–4.44) 0.058
Comorbidity group (*n* = 150)				
OR (95% CI) *p*	2.75 (1.17–6.46) 0.026 *	Undefined	1.95 (0.77–4.94) 0.157	2.09 (0.83–5.23) 0.116

* Represents significant differences.

**Table 5:** Odds ratios of LDs in children according to mode of delivery stratifying by gestational age.

	Unadjusted Results	Premature—Stratum 1 (*n* = 18)	Normal Gestational Age—Stratum 2 (*n* = 238)	Mantel–Haenszel Analysis—Adjusted Results
Main group LDs (*n* = 256)				
OR (95% CI) *p*	1.68 (1.02–2.76) 0.041 *	3.67 (0.35–38.03) 0.261	1.54 (0.92–2.57) 0.096	1.60 (0.97–2.64) 0.065
SLD group (*n* = 186)				
OR (95% CI) *p*	1.16 (0.64–2.11) 0.629	Undefined	1.10 (0.60–2.01) 0.769	1.16 (0.64–2.10) 0.622
ADHD group (*n* = 158)				
OR (95% CI) *p*	2.25 (1.06–4.79) 0.042 *	1.00 (0.08–12.56) 1.000	2.60 (1.14–5.95) 0.021	2.37 (1.09–5.18) 0.030
Comorbidity group (*n* = 150)				
OR (95% CI) *p*	2.75 (1.17–6.46) 0.026 *	Undefined	2.01 (0.82–4.91) 0.122	2.33 (0.99–5.46) 0.048

* Represents significant differences.

**Table 6:** Logistic regression findings about the association between mode of delivery and the occurrence of LDs when adjusting for postnatal–hereditary factors.

Variables		Model
	Unadjusted OR (95% CI) *p*	Adjusted OR (95% CI) *p*
Mode of delivery	1.677 (1.021–2.755) 0.041 *	1.674 (0.966–2.904) 0.066
Breastfeeding	0.453 (0.236–0.869) 0.017 *	n/a
Family history of LDs	4.675 (2.529–8.643) <0.001 **	4.662 (2.460–8.837) <0.001 **
Difficulties in language acquisition	9.490 (3.257–27.657) <0.001 **	9.798 (3.271–29.347) <0.001 **
Language delay	5.127 (2.512–10.466) <0.001 **	n/a

Abbreviations: LDs, learning disabilities; OR, odds ratio; CI, confidence interval; *p*, *p*-value; n/a: not applicable, variables not in the equation. Model adjusted for breastfeeding, family history of LDs, difficulties in language acquisition, and language delay. * Represents significant differences, *p* < 0.05. ** Represents significant differences, *p* < 0.001.

## Data Availability

The original contributions presented in the study are included in the article and Supplementary Materials; further inquiries can be directed to the corresponding author.

## Supplementary Materials

The following supporting information can be downloaded at: https://www.mdpi.com/article/10.3390/children11111386/s1, Table S1: Groups data.

## References

[1] Moll K., Kunze S., Neuhoff N., Bruder J., Schulte-Körne G. (2014). Specific Learning Disorder: Prevalence and Gender Differences. PLoS ONE.

[2] American Psychiatric Association (2013). Diagnostic and Statistical Manual of Mental Disorders: DSM-5.

[3] Cornoldi C., Giofrè D., Orsini A., Pezzuti L. (2014). Differences in the intellectual profile of children with intellectual vs. learning disability. Res. Dev. Disabil..

[4] Tannock R. (2012). Rethinking ADHD and LD in DSM-5: Proposed Changes in Diagnostic Criteria. J. Learn. Disabil..

[5] Görker I. (2019). The Prevalence and Gender Differences in Specific Learning Disorder. Learning Disabilities Neurological Bases, Clinical Features and Strategies of Intervention.

[6] Peterson R.L., Pennington B.F. (2012). Developmental dyslexia. Lancet.

[7] Khodeir M.S., El-Sady S.R., Mohammed H.A.E.-R. (2020). The prevalence of psychiatric comorbid disorders among children with specific learning disorders: A systematic review. Egypt. J. Otolaryngol..

[8] Visser L., Kalmar J., Linkersdörfer J., Görgen R., Rothe J., Hasselhorn M., Schulte-Körne G. (2020). Comorbidities Between Specific Learning Disorders and Psychopathology in Elementary School Children in Germany. Front. Psychiatry.

[9] Frances A. (2013). The new crisis in attention-deficit/hyperactivity disorder diagnosis: Overdiagnosis and overmedication. J. Am. Med. Assoc..

[10] Parens E., Johnston J. (2009). Facts, values, and attention-deficit hyperactivity disorder (ADHD): An update on the controversies. Child Adolesc. Psychiatry Ment. Health.

[11] Willcutt E.G., Pennington B.F., Duncan L., Smith S.D., Keenan J.M., Wadsworth S., Defries J.C., Olson R.K. (2010). Understanding the complex etiologies of developmental disorders: Behavioral and molecular genetic approaches. J. Dev. Behav. Pediatr..

[12] Snowling M.J., Gallagher A., Frith U. (2003). Family Risk of Dyslexia Is Continuous: Individual Differences in the Precursors of Reading Skill. Child Dev..

[13] Lasnick O., Feng J., Quirion A., Hart S., Hoeft F. (2022). The Importance of Family History in Dyslexia. Read. Leag. J..

[14] Larsson H., Chang Z., D’Onofrio B.M., Lichtenstein P. (2013). The heritability of clinically diagnosed attention deficit hyperactivity disorder across the lifespan. Psychol. Med..

[15] Faraone S.V., Larsson H. (2019). Genetics of attention deficit hyperactivity disorder. Mol. Psychiatry.

[16] Richards T.L., Dager S.R., Corina D., Serafini S., Heide A.C., Steury K., Strauss W., Hayes C.E., Abbott R.D., Craft S. (1999). Dyslexic children have abnormal brain lactate response to reading-related language tasks. Am. J. Neuroradiol..

[17] Frank Y., Pavlakis S.G. (2001). Brain imaging in neurobehavioral disorders. Pediatr. Neurol..

[18] Gordon N. (2004). The ‘medical’ investigation of specific learning disorders. J. Pediatr. Neurol..

[19] Morellini L., Ceroni M., Rossi S., Zerboni G., Rege-Colet L., Biglia E., Morese R., Sacco L. (2022). Social Cognition in Adult ADHD: A Systematic Review. Front. Psychol..

[20] Feldman H.M. (2019). The Importance of Language-Learning Environments to Child Language Outcomes. Pediatrics.

[21] Madigan S., Prime H., Graham S.A., Rodrigues M., Anderson N., Khoury J., Jenkins J.M. (2019). Parenting Behavior and Child Language: A Meta-analysis. Pediatrics.

[22] Dy A.B.C., Dy A.B.C., Santos S.K. (2023). Measuring effects of screen time on the development of children in the Philippines: A cross-sectional study. BMC Public Health.

[23] Karbasi Amel A., Rahnamaei H., Hashemi Z. (2023). Play therapy and storytelling intervention on children’s social skills with attention deficit-hyperactivity disorder. J. Educ. Health Promot..

[24] Anderko L., Braun J., Auinger P. (2010). Contribution of Tobacco Smoke Exposure to Learning Disabilities. J. Obstet. Gynecol. Neonatal Nurs..

[25] Eskenazi B., Castorina R. (1999). Association of prenatal maternal or postnatal child environmental tobacco smoke exposure and neurodevelopmental and behavioral problems in children. Environ. Health Perspect..

[26] Gates B., Atherton H. (2007). Causes and manifestations of learning disabilities. Learning Disabilities: Toward Inclusion.

[27] Gao L., Li S., Yue Y., Long G. (2023). Maternal age at childbirth and the risk of attention-deficit/hyperactivity disorder and learning disability in offspring. Front. Public Health.

[28] Saha S., Barnett A.G., Foldi C., Burne T.H., Eyles D.W., Buka S.L., McGrath J.J. (2009). Advanced Paternal Age Is Associated with Impaired Neurocognitive Outcomes during Infancy and Childhood. PLoS Med..

[29] Greene M.M., Patra K., Silvestri J.M., Nelson M.N. (2013). Re-evaluating preterm infants with the Bayley-III: Patterns and predictors of change. Res. Dev. Disabil..

[30] Martínez-Nadal S., Demestre X., Schonhaut L., Muñoz S.R., Sala P. (2018). Impact of neonatal morbidity on the risk of developmental delay in late preterm infants. Early Hum. Dev..

[31] Pierrat V., Marchand-Martin L., Marret S., Arnaud C., Benhammou V., Cambonie G., Debillon T., Dufourg M.-N., Gire C., Goffinet F. (2021). Neurodevelopmental outcomes at age 5 among children born preterm: EPIPAGE-2 cohort study. BMJ.

[32] Rose O., Blanco E., Martinez S.M., Sim E.K., Castillo M., Lozoff B., Vaucher Y.E., Gahagan S. (2013). Developmental Scores at 1 Year with Increasing Gestational Age, 37–41 Weeks. Pediatrics.

[33] Stein R.E.K., Siegel M.J., Bauman L.J. (2006). Are Children of Moderately Low Birth Weight at Increased Risk for Poor Health? A New Look at an Old Question. Pediatrics.

[34] Franz A.P., Bolat G.U., Bolat H., Matijasevich A., Santos I.S., Silveira R.C., Procianoy R.S., Rohde L.A., Moreira-Maia C.R. (2017). Attention-Deficit/Hyperactivity Disorder and Very Preterm/Very Low Birth Weight: A Meta-analysis. Pediatrics.

[35] Breslau N., Johnson E.O., Lucia V.C. (2001). Academic achievement of low birthweight children at age 11: The role of cognitive abilities at school entry. J. Abnorm. Child Psychol..

[36] Kramer M.S., Aboud F., Mironova E., Vanilovich I., Platt R.W., Matush L., Igumnov S., Fombonne E., Bogdanovich N., Ducruet T. (2008). Breastfeeding and child cognitive development: New evidence from a large randomized trial. Arch. Gen. Psychiatry.

[37] Kim J.I., Kim B.-N., Kim J.-W., Hong S.-B., Shin M.-S., Yoo H.J., Cho S.-C. (2017). Breastfeeding is associated with enhanced learning abilities in school-aged children. Child Adolesc. Psychiatry Ment. Health.

[38] Curran E.A., O’Neill S.M., Cryan J.F., Kenny L.C., Dinan T.G., Khashan A.S., Kearney P.M. (2014). Research Review: Birth by caesarean section and development of autism spectrum disorder and attention-deficit/hyperactivity disorder: A systematic review and meta-analysis. J. Child Psychol. Psychiatry.

[39] Zhang T., Sidorchuk A., Sevilla-Cermeño L., Vilaplana-Pérez A., Chang Z., Larsson H., Mataix-Cols D., de la Cruz L.F. (2019). Association of Cesarean Delivery with Risk of Neurodevelopmental and Psychiatric Disorders in the Offspring. JAMA Netw. Open.

[40] Sucksdorff M., Lehtonen L., Chudal R., Suominen A., Gissler M., Sourander A. (2018). Lower Apgar scores and Caesarean sections are related to attention-deficit/hyperactivity disorder. Acta Paediatr..

[41] González-Valenzuela M.-J., López-Montiel D., Cazorla-Granados O., González-Mesa E.-S. (2021). Learning Disabilities in Reading and Writing and Type of Delivery in Twin Births. Children.

[42] Polidano C., Zhu A., Bornstein J.C. (2017). The relation between cesarean birth and child cognitive development. Sci. Rep..

[43] Hanrahan M., McCarthy F.P., O’Keeffe G.W., Khashan A.S. (2019). The association between caesarean section and cognitive ability in childhood. Soc. Psychiatry Psychiatr. Epidemiol..

[44] Curran E.A., Kenny L.C., Dalman C., Kearney P.M., Cryan J.F., Dinan T.G., Khashan A.S. (2017). Birth by caesarean section and school performance in Swedish adolescents- a population-based study. BMC Pregnancy Childbirth.

[45] Blake J.A., Gardner M., Najman J., Scott J.G. (2021). The association of birth by caesarean section and cognitive outcomes in offspring: A systematic review. Soc. Psychiatry Psychiatr. Epidemiol..

[46] Quecke B., Graf Y., Epure A., Santschi V., Chiolero A., Carmeli C., Cullati S. (2021). Caesarean section and obesity in young adult offspring: Update of a systematic review with meta-analysis. Obes. Rev..

[47] Bager P., Wohlfahrt J., Westergaard T. (2008). Caesarean delivery and risk of atopy and allergic disesase: Meta-analyses. Clin. Exp. Allergy.

[48] Koplin J., Allen K., Gurrin L., Osborne N., Tang M.L.K., Dharmage S. (2008). Is caesarean delivery associated with sensitization to food allergens and IgE-mediated food allergy: A systematic review. Pediatr. Allergy Immunol..

[49] Magnus M.C., Haberg S.E., Stigum H., Nafstad P., London S.J., Vangen S., Nystad W. (2011). Delivery by Cesarean Section and Early Childhood Respiratory Symptoms and Disorders: The Norwegian Mother and Child Cohort Study. Am. J. Epidemiol..

[50] Cardwell C.R., Stene L.C., Joner G., Cinek O., Svensson J., Goldacre M.J., Parslow R.C., Pozzilli P., Brigis G., Stoyanov D. (2008). Caesarean section is associated with an increased risk of childhood-onset type 1 diabetes mellitus: A meta-analysis of observational studies. Diabetologia.

[51] Antoniou E., Orovou E., Iliadou M., Sarella A., Palaska E., Sarantaki A., Iatrakis G., Dagla M. (2021). Factors Associated with the Type of Cesarean Section in Greece and Their Correlation with International Guidelines. Acta Inform. Medica.

[52] Snowling M.J., Melby-Lervåg M. (2016). Oral language deficits in familial dyslexia: A meta-analysis and review. Psychol. Bull..

[53] Erbeli F., Hart S.A., Taylor J. (2018). Genetic and Environmental Influences on Achievement Outcomes Based on Family History of Learning Disabilities Status. J. Learn. Disabil..

[54] Thompson P.A., Hulme C., Nash H.M., Gooch D., Hayiou-Thomas E., Snowling M.J. (2015). Developmental dyslexia: Predicting individual risk. J. Child Psychol. Psychiatry.

[55] Puolakanaho A., Ahonen T., Aro M., Eklund K., Leppänen P.H.T., Poikkeus A.-M., Tolvanen A., Torppa M., Lyytinen H. (2007). Very early phonological and language skills: Estimating individual risk of reading disability. J. Child Psychol. Psychiatry.

[56] Grimm O., Kranz T.M., Reif A. (2020). Genetics of ADHD: What Should the Clinician Know?. Curr. Psychiatry Rep..

[57] Storch S.A., Whitehurst G.J. (2002). Oral language and code-related precursors to reading: Evidence from a longitudinal structural model. Dev. Psychol..

[58] Bishop D.V.M., Snowling M.J. (2004). Developmental dyslexia and specific language impairment: Same or different?. Psychol. Bull..

[59] Hawkins E., Gathercole S., Astle D., Holmes J., The CALM Team (2016). Language Problems and ADHD Symptoms: How Specific Are the Links?. Brain Sci..

[60] Green B.C., Johnson K.A., Bretherton L. (2013). Pragmatic language difficulties in children with hyperactivity and attention problems: An integrated review. Int. J. Lang. Commun. Disord..

[61] Spyrakou E., Magriplis E., Benetou V., Zampelas A. (2022). Factors Associated with Breastfeeding Initiation and Duration in Greece: Data from the Hellenic National Nutrition and Health Survey. Children.

[62] Akhutina T.V., Pylaeva N.M. (2011). L. Vygotsky, A. Luria and Developmental Neuropsychology. Psychol. Russ. State Art.

